# Chemical composition of copra, palm kernel, and cashew co-products from South-East Asia and almond hulls from Australia

**DOI:** 10.5713/ab.22.0359

**Published:** 2023-02-26

**Authors:** Natalia S. Fanelli, Leidy J. Torres-Mendoza, Jerubella J. Abelilla, Hans H. Stein

**Affiliations:** 1 Department of Animal Sciences, University of Illinois, Urbana, IL 61801, USA; 2 DSM Nutritional Products, Mapletree Business City 117440, Singapore

**Keywords:** Almond Hulls, Alternative Feed Ingredient, Cashew Nut, Chemical Composition, Copra, Palm Kernel

## Abstract

**Objective:**

Oilseeds and nut co-products can be used as alternative feed ingredients in animal diets because they may have a lower cost than traditional ingredients. A study was, therefore, conducted to determine the chemical composition of copra, palm kernel, and nut co-products from South-East Asia or Australia. The hypothesis that country of production influences nutritional composition was tested.

**Methods:**

Oilseed meals included 2 copra expellers, 3 copra meals, and 12 palm kernel expellers. One source of almond hulls and cashew nut meal were also used. Samples were obtained from suppliers located in South-East Asia or Australia. All samples were analyzed for dry matter, gross energy, nitrogen, amino acids (AA), acid-hydrolyzed ether extract (AEE), ash, minerals, insoluble dietary fiber, and soluble dietary fiber. Copra and nut co-products were also analyzed for total starch and sugars.

**Results:**

Copra expellers had greater (p<0.05) concentrations of dry matter and AEE compared with copra meal. However, copra meal had greater (p<0.05) concentrations of total dietary fiber (soluble and insoluble) and copper than copra expellers. Palm kernel expellers from Indonesia had greater (p<0.05) concentration of histidine and tyrosine compared with palm kernel expellers from Vietnam. Almond hulls was high in dietary fiber, but also contained free glucose and fructose, whereas cashew nut meal was high in AEE, but low in all free sugars.

**Conclusion:**

Copra expellers have greater concentration of AEE, but less concentration of total dietary fiber when compared with copra meal, and except for a few AA, no differences in nutrient composition of palm kernel expellers produced in Indonesia or Vietnam were detected. According to the chemical composition of nut co-products, cashew nut meal may be more suitable for non-ruminant diets than almond hulls.

## INTRODUCTION

Global livestock and poultry production is increasing, which has increased the demand for cereal grains, oilseed meals, and alternative feed ingredients for animal feeding [1]. Fibrous crop residues have traditionally been used in Asia [2], but a variety of oilseed meals are available and can be used in animal diets. Copra meal, a co-product of copra, has been used in South-East Asia as a low-cost ingredient [2]. Copra co-products consist of the dried and ground residue that remains after removal of most of the oil from the coconut. If the oil is removed by a mechanical process, the co-product that is left is called copra expellers, and if oil is removed using solvent extracts, the remaining co-product is known as copra meal [3,4]. Co-products from the palm kernel, an oil-rich endosperm in the hard endosperm of palm, is also used in the feed industry [3]. After the palm oil is mechanically extracted, the main co-product is known as palm kernel expellers, which can be used as protein and energy source in animal diets [5].

Nut-coproducts have been used to partially replace energy or protein in diets for livestock. Almond hulls consist of the fleshy mesocarp and pericarp of the fruit, which splits open when mature and accounts for approximately 52% of the total weight of the fruit [6]. Almonds are harvested by smashing and collecting the fruits with self-propelled machines [7]. Following collection, the fruits are dried and screened to remove impurities, and the almond kernels and shells are separated [7]. Cashew nuts, also known as cashew kernels, are harvested when mature and fruits are separated. The most valuable product is the nut, which must be extracted from its shell and roasted to destroy toxins in the shell oil [8]. Discarded cashew nuts are sold as cashew nut meal.

It is important to characterize the composition of copra co-products, palm kernel expellers, almond hulls, and cashew nut meal as possible alternative feed ingredients that can be used as substitutes for conventional ingredients in animal diets. However, there is limited information about the full chemical composition of copra, palm kernel, and nut co-products that are available in South-East Asia or Australia, with most studies focusing on a few nutrients from a specific location. Furthermore, information on if the analyzed components add up to 100% is not always available [3,9,10]. Therefore, the objectives of this study were to determine the chemical composition of copra, palm kernel, and cashew nut co-products from South-East Asia and almond hulls from Australia. The hypothesis was that there are differences between copra meal and copra expellers and that the composition of palm kernel expellers produced in different countries is not different.

## MATERIALS AND METHODS

### Description of samples

Sources of copra, palm kernel, and cashew nut co-products from feed mills in South-East Asia, and a source of almond hulls from Australia were delivered to DSM Nutritional Products, Singapore. The suppliers provided between 100 and 300 grams of each ingredient. Samples were labeled, cataloged, and then shipped to the University of Illinois, Urbana, IL, USA, where most of the chemical analyses were conducted. Oilseed coproducts included two sources of copra expellers from the Philippines, two sources of copra meal from the Philippines, and one source of copra meal from Vietnam. Twelve sources of palm kernel expellers from Indonesia, the Philippines, and Vietnam were also used. Nut co-products included one source of almond hulls from Australia and one cashew nut meal from Indonesia.

### Chemical analysis

Samples of all feed ingredients were finely ground and analyzed for dry matter (method 930.15) [11] and ash (method 942.05) [11]. Gross energy was analyzed using an isoperibol bomb calorimeter (model 6400; Parr Instruments, Moline, IL, USA). Samples were analyzed for amino acids (AA; method 982.30 E a, b, and c) [11] on a Hitachi AA Analyzer (Model L8800; Hitachi High Technologies America Inc., Pleasanton, CA, USA) and nitrogen was analyzed by combustion (method 990.03) [11] using a LECO FP628 Nitrogen Analyzer (LECO Corp., Saint Joseph, MI, USA). Crude protein was calculated as nitrogen×6.25. Acid-hydrolyzed ether extract (AEE) was analyzed using 3N HCl (Ankom^HCl^; Ankom Technology, Macedon, NY, USA) followed by crude fat extraction using petroleum ether (Ankom^XT15^; Ankom Technology, USA). Insoluble and soluble dietary fiber were quantified according to method 991.43 [11] using the Ankom^TDF^ Dietary Fiber Analyzer (Ankom Technology, USA). Total dietary fiber was calculated as the sum of insoluble and soluble dietary fiber. Minerals were analyzed (method 985.01 a, b, and c) [11] using inductively coupled plasma-optical emission spectrometry (ICP-OES; Avio 200; PerkinElmer, Waltham, MA, USA). Sample preparation included dry ashing at 600°C for 4 h (method 942.05; 10) [11] and wet digestion with nitric acid (method 3050 B) [12]. Total starch was analyzed in the copra and nut co-products using the glucoamylase procedure (method 979.10) [11]. Sugars including glucose, fructose, maltose, sucrose, stachyose, and raffinose were also analyzed in copra and nut co-products using high-performance liquid chromatography (Dionex App Notes 21 and 92).

### Calculations and statistical analysis

For each analysis of copra and nut co-products, analyzed proximate components were added and subtracted from the concentration of dry matter in each ingredient to calculate the un-analyzed rest fraction according to the following equation:


$$\begin{array}{l} \text{Rest fraction}=[\text{dry matter}-(\text{crude protein}+\text{AEE}+\text{ash}\\ +\text{total dietary fiber}+\text{total starch}+\text{glucose}+\text{fructose}+\\ \text{maltose}+\text{sucorse}+\text{stachyose}+\text{raffinose})]. \end{array}$$

The rest fraction for palm kernel expellers was calculated using the same equation, with the exception that total starch and sugars were not analyzed in palm kernel expellers and, therefore, not included in the equation.

The coefficient of variation and average concentration of nutrients in samples within each group of feed ingredients were calculated if two or more samples from the same country were available. Normality of residues and homogeneity of variances were verified using the UNIVARIATE procedure (SAS 9.4 Institute Inc. Cary, NC, USA). Data were analyzed by analysis of variance using the PROC MIXED procedure in SAS to test statistical differences between copra co-products and between country of origin for palm kernel expellers. The replicate sample was the experimental unit for all analyses. The feed ingredient or country was the fixed effect, and the replicate sample was the random effect. Means were calculated using the LSMEANS statement in SAS, and when significant, means were separated using the PDIFF option in the MIXED procedure. Results were considered significant at p<0.05.

## RESULTS AND DISCUSSION

### Copra and palm kernel co-products

The chemical composition of copra co-products (Tables 1 and 2) and palm kernel expellers (Tables 3 and 4) were within the range of published values for these ingredients [3,13–15]. The analyzed components in the chemical composition of copra co-products and palm kernel expellers were close to 100%, indicating that all nutrients in these ingredients were accounted for [13]. This is indicated by the fact that the calculated rest fraction was close to or less than 3% for all samples. Except for dry matter, all nutrient analysis results were adjusted to 88% dry matter because this is a typical value for oilseeds and allows for a direct comparison without the influence of moisture.

Copra expellers had greater (p <0.05) concentrations of dry matter and AEE compared with copra meal. However, copra meal had greater (p<0.05) concentrations of total dietary fiber (soluble and insoluble) and copper than copra expellers. The differences in nutrient composition between copra expellers and copra meal are primarily due to differences in residual oil remaining in the product [3], and the nutritional quality of oilseed meals is dependent on the process of oil extraction [1]. Copra meal samples from Vietnam and the Philippines appeared to be very similar in composition.

No differences were observed between the palm kernel expellers from Indonesia and Vietnam, with the exception of histidine and tyrosine, which were greater (p<0.05) in palm expellers from Indonesia compared with palm kernel expellers from Vietnam. The observation that there were few differences in AA concentration between countries for palm kernel expellers indicates that the growing conditions in Indonesia and Vietnam did not have major impacts on composition. It also appears that the processing was not different between the two countries.

Both copra and palm kernel co-products are used as protein sources in animal diets, but their protein content is relatively low when compared with other oilseed meals, with approximately 21.50% in copra co-products and 15.50% in palm kernel expellers. Calculated lysine/crude protein ratio for samples in this study was on average 2.30% and 2.70% for copra co-products and palm kernel expellers, respectively. However, arginine is the indispensable AA present in the greatest concentration in both co-products with calculated arginine/crude protein ratios of 9.80% and 10.70% for copra co-products and palm kernel expellers, respectively. The high concentration of arginine (2% for copra co-products and 1.70% for palm kernel expellers) may result in antagonism because arginine competes for the same transporter in the enterocytes as lysine [16]. As a result, adequate levels of digestible lysine in diets are required to mitigate the negative effects of the high arginine content of these co-products [3].

Total dietary fiber concentrations in copra expellers aver aged 42%, 47% in copra meal, and approximately 60% in palm kernel expellers, and these values are consistent with published data [3]. Because of the high fiber content, palm kernel expellers may not be suitable for feeding of young pigs and poultry [5]. However, with adequate levels of standardized ileal digestible AA, inclusion of up to 10% palm kernel expellers may be acceptable for weanling pigs [3]. Inclusion of more than 10% of copra co-products in diets for weanling pigs reduced average daily feed intake, most likely due to the slow rate of passage of the fiber through the digestive tract, which results in increased gut fill and therefore reduced feed intake [17].

When compared with other oilseed meals, except soybean meal, copra co-products has a high concentration of sucrose, with around 7%, but unlike soybean meal and other legumes, copra co-products do not contain oligosaccharides that may increase diarrhea in young animals [18,19]. The sugar profile of copra co-products determined in this study is in accordance with data for copra expellers [13]. Because of the high sucrose content in copra co-products, digestible and metabolizable energy values are greater in copra co-products than in palm kernel expellers [3]. The sucrose concentration in copra meal is most likely due to the sucrose in the coconut sap, which transports nutrients to the coconut [20].

The mineral composition demonstrated that potassium was present in high concentration in both co-products, with approximately 2.10% in copra co-products and 0.64% in palm kernel expellers, but mineral concentration in feed ingredients may be influenced by soil mineral composition [21]. Copra and palm kernel co-products were also high in phosphorus and iron, with an average of 0.62% phosphorus and 600 mg/kg iron, with the level of phosphorus being close to published values for soybean meal. These values are also in agreement with previously reported values for copra meal and palm kernel expellers [22]. Less than one-third of the phosphorus in copra co-products is bound to phytate, whereas the majority of the phosphorus in palm kernel expellers is bound to phytate [22]. Therefore, standardized total tract digestibility of phosphorus in diets containing palm kernel expellers can be improved if phytase is added [22].

### Nut co-products

The chemical composition of almond hulls and cashew nut meal (Tables 5 and 6) were in agreement with published values for these ingredients [7,8,23,24]. Except for dry matter, all analyzed nutrients were adjusted to 88% dry matter to allow for a direct comparison of chemical composition among ingredients. The analyzed components in almond hulls added up to close to 100%, indicating that all nutrients for this ingredient were accounted for [13]. In contrast, despite extensive nutrient analysis of cashew nut meal, analyzed nutrients accounted for only 94.50% of this ingredient, and it is not clear what the remaining 5.50% consist of. It is, however, possible that additional carbohydrate fractions that were not captured in the analyzed carbohydrate components, account for the remaining nutrients. Nevertheless, AEE represented a significant part of the analyzed nutrients in cashew nut meal, although at a lower concentration in this study compared with previous data [8]. The cashew nut meal contained approximately 35% AEE, which is due to the fact that fresh raw cashew kernels contain approximately 48% AEE, however, the nutritional composition of processed cashew nut meal is influenced to some extent by the region where the cashew trees are grown [25].

The nutrient composition of cashew nut meal analyzed in this study also indicated a high concentration of AA, with approximately 8% being indispensable AA and a lysine/crude protein ratio of 4.50%, which is very favorable for pigs and poultry. The 24% total dietary fiber in the cashew nut meal will likely not be a problem in diets for pigs and poultry, and the AEE concentration will increase energy in the diets. Cashew nut meal may partially replace corn in swine diets [26], and can also be included in broiler diets at an inclusion rate of 20% to 25% without affecting growth performance [10].

Almond hulls is a co-product with some variation in com position among different sources, and the nutritional quality is determined by cultivation, variety, processing, and harvesting conditions [7]. Analyzed composition of almond hulls in this study demonstrated that sugar, primarily glucose, fructose, and sucrose, accounted for nearly 23% of total nutrient concentration. This is generally in agreement with reported values for almond hulls, although sucrose was slightly lower in this study than reported by Sequeira and Lew [27] and DePeters et al [9]. Rain-damage may reduce sugar concentration, and thus energy values in almond hulls [7]. Very high concentration of iron was also observed in both nut co-products, with approximately 500 mg/kg in almond hulls and 750 mg/kg in cashew nut meal.

The analyzed chemical composition demonstrated that almond hulls contained around 44% total dietary fiber, and almond hulls may, therefore, be more suitable for ruminants than for pigs and poultry. Due to its fiber and energy contents, almond hulls can be used as a forage or as a concentrate ingredient for ruminants [28], with inclusion levels limited to less than 30% in diets for growing steers [29]. Diets containing almond hulls also need to be supplemented with extra protein due to the low protein concentration in almond hulls (approximately 5%). Calvert and Parker [30] demonstrated that 10% to 15% conventional grain may be replaced with almond hulls in diets for growing pigs if protein levels are balanced.

## CONCLUSION

The chemical composition of copra co-products demonstrated that copra expellers have greater concentration of AEE, but less concentration of total dietary fiber compared with copra meal. Despite minor differences in AA, palm kernel expellers produced in Indonesia generally have a chemical composition that is not different from palm kernel expellers produced in Vietnam. According to the chemical composition of nut co-products, cashew nut meal may be more suitable in diets for non-ruminant animals due to the lower fiber content when compared with almond hulls.

## Figures and Tables

**Table 1:** Analyzed nutrient composition of copra expellers and copra meal^1)^

Item (%)	Philippines								Vietnam	Expellers vs meal	
	
	Copra expellers				Copra meal					SEM	p-value
					v						
	Sample 1	Sample 2	CV	Average	Sample 1	Sample 2	CV	Average			
Dry matter	94.46	92.74	1.30	93.60	84.39	88.74	3.55	86.57	87.37	1.26	0.032
Gross energy (kcal/kg)	4,108	4,143	0.59	4,126	3,775	3,908	2.45	3,841	3,897	42.39	0.060
Crude protein	20.86	20.87	0.02	20.86	21.47	21.80	1.07	21.63	21.98	0.21	0.209
AEE	7.32	7.42	0.94	7.37	1.61	2.71	36.11	2.16	2.72	0.38	0.022
Ash	5.89	5.94	0.62	5.91	6.21	6.25	0.37	6.23	6.68	0.21	0.097
Carbohydrates											
Total starch	1.32	2.02	29.53	1.67	1.67	1.32	16.55	1.49	1.32	0.21	0.495
Insoluble dietary fiber	37.64	38.05	0.77	37.84	41.82	41.15	1.13	41.48	41.40	0.21	0.001
Soluble dietary fiber	3.54	4.93	23.27	4.24	5.01	6.45	17.79	5.73	4.83	0.51	0.011
Total dietary fiber	41.18	42.98	3.04	42.08	46.82	47.60	1.17	47.21	46.23	0.58	0.048
Glucose	0.20	0.15	17.83	0.17	0.28	0.20	24.52	0.24	0.35	0.04	0.144
Fructose	0.83	0.61	21.84	0.72	0.23	0.82	79.77	0.53	1.08	0.24	0.978
Maltose	ND	ND	-	-	ND	ND	-	-	ND	-	-
Sucrose	8.08	4.89	34.80	6.48	6.48	8.08	15.61	7.28	7.45	0.94	0.568
Stachyose	ND	ND	-	-	ND	ND	-	-	ND	-	-
Raffinose	ND	ND	-	-	ND	ND	-	-	ND	-	-
Rest fraction^2)^	2.33	3.12	-	2.73	3.03	−0.77	-	1.13	0.19	1.06	0.294
Minerals											
Calcium	0.07	0.09	18.96	0.08	0.09	0.08	11.85	0.09	0.09	0.01	0.495
Phosphorus	0.58	0.62	4.64	0.60	0.64	0.61	2.40	0.63	0.66	0.02	0.223
Magnesium	0.29	0.33	9.86	0.31	0.32	0.33	0.87	0.33	0.34	0.01	0.383
Potassium	1.96	2.05	3.29	2.00	2.34	2.14	6.12	2.24	2.13	0.07	0.127
Sodium	0.05	0.07	24.83	0.06	0.05	0.07	20.10	0.06	0.02	0.01	0.556
Sulfur	0.09	0.09	6.15	0.09	0.10	0.09	10.98	0.10	0.10	0.00	0.219
Copper (mg/kg)	25.43	30.05	11.77	27.74	34.87	33.37	3.11	34.12	33.25	1.31	0.046
Iron (mg/kg)	298.76	613.46	48.79	456.11	251.28	391.06	30.78	321.17	548.31	112.01	0.426
Manganese (mg/kg)	72.04	74.28	2.16	73.16	80.27	68.70	10.98	74.49	74.93	3.11	0.760
Zinc (mg/kg)	39.31	41.55	3.91	40.43	43.82	43.81	0.01	43.81	50.18	2.02	0.150

CV, coefficient of variation; SEM, standard error of the means; AEE, acid-hydrolyzed ether extract; ND, not detected.

1) Except for dry matter, all values were adjusted to 88% dry matter.

2) Rest fraction = calculated using the following equation: [dry matter − (crude protein + AEE + ash + total dietary fiber + total starch + glucose + fructose + maltose + sucrose + stachyose + raffinose)].

**Table 2:** Analyzed amino acid composition of copra expellers and copra meal^1)^

Item (%)	Philippines								Vietnam	Expellers vs meal	
	
	Copra expellers				Copra meal					SEM	p-value
	
	Sample 1	Sample 2	CV	Average	Sample 1	Sample 2	CV	Average			
Indispensable AA											
Arginine	2.00	2.15	5.14	2.08	2.09	2.13	1.56	2.11	1.95	0.07	0.826
Histidine	0.39	0.38	2.15	0.39	0.39	0.36	5.49	0.37	0.37	0.01	0.401
Isoleucine	0.70	0.72	2.24	0.71	0.73	0.74	1.32	0.74	0.71	0.01	0.137
Leucine	1.27	1.28	0.78	1.27	1.28	1.27	0.74	1.28	1.23	0.01	0.509
Lysine	0.45	0.47	4.18	0.46	0.56	0.50	8.98	0.53	0.50	0.02	0.112
Methionine	0.26	0.28	6.17	0.27	0.28	0.29	1.50	0.28	0.28	0.01	0.162
Phenylalanine	0.91	0.89	1.65	0.90	0.89	0.91	2.04	0.90	0.86	0.01	0.556
Threonine	0.61	0.63	1.30	0.62	0.64	0.60	3.55	0.62	0.61	0.01	0.859
Tryptophan	0.12	0.16	20.13	0.14	0.15	0.15	1.32	0.15	0.14	0.01	0.696
Valine	1.02	1.02	0.00	1.02	1.01	1.03	1.37	1.02	0.96	0.02	0.511
Total	7.73	7.98	2.25	7.86	8.02	7.98	8.00	0.35	7.61	0.14	0.943
Dispensable AA											
Alanine	0.82	0.84	1.30	0.83	0.81	0.85	3.35	0.83	0.85	0.01	0.900
Aspartic acid	1.56	1.60	2.14	1.58	1.63	1.62	0.45	1.62	1.60	0.01	0.146
Cysteine	0.31	0.32	3.41	0.32	0.35	0.33	5.66	0.34	0.34	0.01	0.058
Glutamic acid	3.34	3.47	2.86	3.40	3.55	3.61	1.27	3.58	3.60	0.04	0.071
Glycine	0.84	0.86	2.08	0.85	0.91	0.86	3.55	0.88	0.88	0.01	0.197
Proline	0.69	0.67	1.63	0.68	0.70	0.68	1.47	0.69	0.72	0.01	0.316
Serine	0.75	0.78	3.04	0.76	0.79	0.73	5.44	0.76	0.78	0.02	0.954
Tyrosine	0.32	0.46	25.40	0.39	0.45	0.44	1.93	0.44	0.47	0.04	0.321
Total	8.63	9.00	2.97	8.82	9.19	9.12	0.54	9.16	9.24	0.10	0.085
Total AA	16.36	16.98	2.63	16.67	17.21	17.10	0.45	17.16	16.85	0.19	0.249

CV, coefficient of variation; SEM, standard error of the means; AA, amino acids.

1) All values were adjusted to 88% dry matter.

**Table 3:** Analyzed nutrient composition of palm kernel expellers^1)^

Item (%)	Indonesia				Philippines	Vietnam											Indonesia vs Vietnam	
		
	Sample 1	Sample 2	CV	Average		Sample 1	Sample 2	Sample 3	Sample 4	Sample 5	Sample 6	Sample 7	Sample 8	Sample 9	CV	Average	SEM	p-value
Dry matter	92.58	92.79	0.16	92.69	92.82	92.78	93.16	93.48	90.92	91.78	91.57	91.24	92.15	92.86	0.97	92.22	0.30	0.190
GE (kcal/kg)	4,172	4,295	2.06	4,234	4,225	4,347	4,188	4,103	4,267	4,084	4,286	4,764	4,545	4,419	5.03	4,334	114.84	0.552
Crude protein	16.06	16.06	0.03	16.06	14.84	13.71	15.29	15.94	14.85	16.61	15.59	15.02	16.60	17.16	6.83	15.64	0.56	0.607
AEE	8.53	8.45	0.63	8.49	7.80	8.45	7.30	5.87	8.45	5.28	8.54	9.26	7.92	6.33	18.38	7.49	0.61	0.478
Ash	4.87	3.79	17.53	4.33	4.02	3.68	3.46	5.60	4.05	3.91	3.86	4.03	3.75	6.03	21.21	4.26	0.47	0.475
Carbohydrates																		
IDF	54.18	55.48	1.68	54.83	56.69	59.28	57.15	52.91	57.01	58.68	56.51	64.97	62.02	56.97	5.94	58.39	1.82	0.199
SDF	3.42	2.94	10.71	3.18	2.94	2.28	3.21	3.29	2.03	2.30	3.17	2.49	3.49	3.55	20.45	2.87	0.31	0.497
TDF	57.60	58.42	1.00	58.01	59.63	61.56	60.36	56.20	59.04	60.98	59.68	67.47	65.50	60.52	5.51	61.26	1.76	0.225
Rest fraction^2)^	0.94	1.28	-	1.11	1.71	0.61	1.59	4.39	1.62	1.22	0.88	0.67	1.72	4.72	-	1.94	0.55	0.948
Minerals																		
Calcium	0.81	0.27	71.46	0.54	0.30	0.27	0.42	0.56	0.55	0.44	0.56	0.29	0.45	0.60	26.04	0.46	0.09	0.562
Phosphorus	0.67	0.56	12.22	0.61	0.61	0.56	0.65	0.58	0.62	0.66	0.58	0.61	0.71	0.63	7.53	0.62	0.03	0.861
Magnesium	0.32	0.28	11.38	0.30	0.29	0.28	0.29	0.34	0.28	0.29	0.26	0.30	0.32	0.36	10.56	0.30	0.02	0.930
Potassium	0.63	0.66	4.00	0.65	0.62	0.58	0.66	0.69	0.65	0.65	0.60	0.63	0.72	0.74	7.96	0.66	0.03	0.692
Sodium	0.01	0.01	0.16	0.01	0.01	0.01	0.01	0.02	0.01	0.02	0.01	0.01	0.01	0.02	37.50	0.01	0.00	0.389
Sulfur	0.06	0.04	28.44	0.05	0.04	0.03	0.04	0.06	0.05	0.05	0.05	0.03	0.04	0.06	24.81	0.05	0.01	0.637
Copper (mg/kg)	40.81	35.63	9.58	38.22	25.00	23.08	33.66	46.69	24.23	28.84	24.25	25.29	36.52	50.28	31.22	32.54	5.34	0.471
Iron (mg/kg)	355.63	999.11	67.17	677.37	751.16	1,031.60	213.81	724.08	601.80	502.71	688.74	1,130.64	232.03	779.75	47.87	656.13	183.90	0.937
Manganese (mg/kg)	297.50	186.71	32.36	242.10	278.90	263.36	160.40	316.16	284.73	304.64	220.59	288.65	174.06	340.47	24.15	261.45	21.33	0.082
Zinc (mg/kg)	41.93	39.78	3.71	40.86	38.43	36.89	42.69	41.45	39.37	48.66	40.21	40.43	46.32	44.64	8.71	42.30	1.94	0.612

CV, coefficient of variation; SEM, standard error of the means; GE, gross energy; AEE, acid-hydrolyzed ether extract; IDF, insoluble dietary fiber; SDF, soluble dietary fiber; TDF, total dietary fiber.

1) Except for dry matter, all values were adjusted to 88% dry matter.

2) Rest fraction = calculated using the following equation: [dry matter − (crude protein + AEE + ash + total dietary fiber + total starch)].

**Table 4:** Analyzed amino acid composition of palm kernel expellers^1)^

Item (%)	Indonesia				Philippines	Vietnam											Indonesia vs Vietnam	
		
	Sample 1	Sample 2	CV	Average		Sample 1	Sample 2	Sample 3	Sample 4	Sample 5	Sample 6	Sample 7	Sample 8	Sample 9	CV	Average	SEM	p-value
Indispensable AA																		
Arginine	1.95	1.59	14.19	1.77	1.70	1.47	1.50	1.29	1.46	1.78	1.42	1.61	1.63	1.39	9.75	1.51	0.09	0.067
Histidine	0.29	0.27	7.35	0.28	0.25	0.23	0.23	0.23	0.22	0.28	0.21	0.25	0.25	0.24	8.61	0.24	0.01	0.024
Isoleucine	0.58	0.58	0.16	0.58	0.54	0.49	0.49	0.50	0.50	0.61	0.53	0.54	0.53	0.54	7.19	0.53	0.02	0.082
Leucine	1.00	1.00	0.16	1.00	0.93	0.84	0.84	0.88	0.89	1.04	0.89	0.93	0.91	0.95	6.79	0.91	0.03	0.073
Lysine	0.50	0.39	18.21	0.45	0.44	0.41	0.37	0.29	0.33	0.45	0.32	0.45	0.40	0.31	16.38	0.37	0.03	0.345
Methionine	0.30	0.29	2.40	0.30	0.28	0.26	0.26	0.25	0.26	0.30	0.27	0.28	0.28	0.27	5.56	0.27	0.01	0.053
Phenylalanine	0.66	0.65	0.16	0.66	0.63	0.57	0.51	0.56	0.59	0.67	0.60	0.62	0.55	0.61	7.86	0.59	0.03	0.219
Threonine	0.46	0.45	1.65	0.45	0.45	0.40	0.39	0.39	0.43	0.46	0.41	0.44	0.42	0.42	5.59	0.42	0.01	0.060
Tryptophan	0.10	0.10	6.57	0.10	0.10	0.07	0.09	0.09	0.10	0.11	0.10	0.07	0.10	0.10	15.12	0.09	0.01	0.469
Valine	0.82	0.82	0.16	0.82	0.74	0.67	0.69	0.72	0.73	0.85	0.74	0.74	0.75	0.77	6.92	0.74	0.03	0.063
Total	6.66	6.14	5.75	6.40	6.06	5.41	5.37	5.20	5.51	6.55	5.49	5.93	5.82	5.60	7.81	5.65	0.29	0.260
Dispensable AA																		
Alanine	0.62	0.63	0.92	0.62	0.58	0.53	0.53	0.56	0.56	0.65	0.57	0.58	0.57	0.61	6.58	0.57	0.02	0.097
Aspartic acid	1.22	1.19	1.84	1.20	1.17	1.04	1.01	1.02	1.10	1.25	1.11	1.14	1.10	1.09	6.63	1.10	0.04	0.073
Cysteine	0.23	0.20	9.59	0.21	0.22	0.18	0.18	0.17	0.19	0.22	0.20	0.20	0.19	0.18	7.89	0.19	0.01	0.074
Glutamic acid	2.95	2.82	3.19	2.88	2.65	2.37	2.39	2.50	2.56	3.00	2.53	2.60	2.59	2.70	7.25	2.58	0.10	0.059
Glycine	0.69	0.66	3.13	0.68	0.65	0.59	0.59	0.61	0.63	0.72	0.62	0.64	0.64	0.66	6.32	0.63	0.02	0.198
Proline	0.48	0.46	2.99	0.47	0.48	0.44	0.42	0.42	0.44	0.51	0.42	0.48	0.45	0.46	6.82	0.45	0.02	0.380
Serine	0.59	0.55	4.87	0.57	0.56	0.51	0.50	0.48	0.54	0.58	0.50	0.56	0.54	0.52	6.10	0.53	0.02	0.189
Tyrosine	0.32	0.30	4.45	0.31	0.29	0.23	0.24	0.27	0.26	0.29	0.27	0.25	0.26	0.29	7.81	0.26	0.01	0.013
Total	7.10	6.81	2.95	6.96	6.60	5.89	5.86	6.03	6.28	7.22	6.22	6.45	6.34	6.51	6.52	6.31	0.17	0.088
Total AA	13.76	12.95	4.29	13.36	12.66	11.30	11.23	11.23	11.79	13.77	11.71	12.38	12.16	12.11	6.68	11.96	0.41	0.154

CV, coefficient of variation; SEM, standard error of the means; AA, amino acids.

1) All values were adjusted to 88% dry matter.

**Table 5:** Analyzed nutrient composition of nut co-products^1)^

Item (%)	Australia	Indonesia
	
	Almond hulls	Cashew nut meal
Dry matter	85.51	90.97
Gross energy (kcal/kg)	3,836	5,641
Crude protein	4.46	18.06
AEE	2.07	34.77
Ash	8.07	2.89
Carbohydrates		
Total starch	2.37	ND
Insoluble dietary fiber	36.23	19.83
Soluble dietary fiber	7.51	3.58
Total dietary fiber	43.74	23.41
Glucose	9.96	0.59
Fructose	9.29	0.22
Maltose	0.12	0.15
Sucrose	4.00	1.37
Stachyose	ND	0.28
Raffinose	ND	0.90
Rest fraction^2)^	3.92	5.35
Minerals		
Calcium	0.23	0.08
Phosphorus	0.15	0.46
Magnesium	0.08	0.23
Potassium	3.26	0.69
Sodium	0.03	0.02
Sulfur	0.06	0.03
Copper (mg/kg)	25.51	23.98
Iron (mg/kg)	461.84	731.48
Manganese (mg/kg)	26.65	27.31
Zinc (mg/kg)	33.15	56.54

AEE, acid-hydrolyzed ether extract; ND, not detected.

1) Except for dry matter, all values were adjusted to 88% dry matter.

2) Rest fraction = calculated using the following equation: [Dry matter − (crude protein + AEE + ash + total dietary fiber + total starch + glucose + fructose + maltose + sucrose + stachyose + raffinose)].

**Table 6:** Analyzed amino acid composition of nut co-products^1)^

Item (%)	Australia	Indonesia
	
	Almond hulls	Cashew nut meal
Indispensable AA		
Arginine	0.13	1.65
Histidine	0.08	0.38
Isoleucine	0.13	0.78
Leucine	0.22	1.28
Lysine	0.15	0.81
Methionine	0.04	0.31
Phenylalanine	0.12	0.85
Threonine	0.12	0.63
Tryptophan	0.02	0.06
Valine	0.16	1.00
Total	1.19	7.75
Dispensable AA		
Alanine	0.16	0.71
Aspartic acid	0.65	1.60
Cysteine	0.05	0.36
Glutamic acid	0.34	3.18
Glycine	0.15	0.76
Proline	0.23	0.68
Serine	0.15	0.79
Tyrosine	0.06	0.57
Total	1.80	8.65
Total AA	2.99	16.40

AA, amino acids.

1) All values were adjusted to 88% dry matter.
